# From Xeroderma Pigmentosum to malignant melanoma: case of an 8-year-old boy

**DOI:** 10.1093/omcr/omag027

**Published:** 2026-03-23

**Authors:** Muhammad Usama Bin Shabbir, Faiza Fatima, Fatima Hussain, Sikandar Ajmal Abbasi, Gohar Ali, Hareem Binte Saleem, Raghabendra Kumar Mahato

**Affiliations:** General Medicine, Pakistan Institute of Medical Sciences, Islamabad, Pakistan; Services Institute of Medical Sciences, Lahore, Pakistan; Rawalpindi Medical University, Rawalpindi, Pakistan; Tehsil Headquarters Hospital, Murree, Pakistan; General Medicine, Pakistan Institute of Medical Sciences, Islamabad, Pakistan; Memorial HealthCare, Hollywood, Florida; Gandaki Medical College Teaching Hospital and Research Center, Pokhara, Nepal

**Keywords:** melanoma, Xeroderma Pigmentosum, skin carcinoma, hyperpigmentation

Xeroderma Pigmentosum (XP) is a predominantly autosomal recessive disorder, characterized by impaired nucleotide excision repair following ultraviolet-induced DNA damage [1]. XP is associated with an increased risk for development of basal cell carcinoma and squamous cell carcinoma at an early age [2, 3]. Malignant melanoma, although quite rare in childhood, is one of the serious complications of XP, and presents aggressively with an extremely poor prognosis if not promptly treated [4, 5].

**Figure 1:**
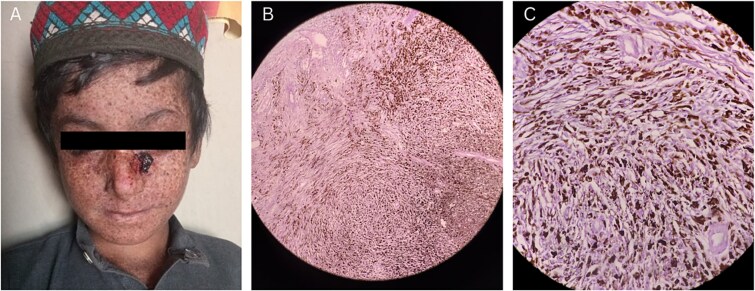
(A) Image showing an irregular, nodular and erythematous lesion on the bridge of nose. (B) Histopathology revealing an epithelioid tumour arranged in the form of nests and sheets showing melanocytic differentiation. (C) Histopathology revealing spindle shaped cells with prominent nucleoli.

An eight-year-old boy presented to the out-patient department with the history of abnormal freckling on sun-exposed skin areas (e.g. face, hands and neck), severe sunburns and redness upon minimal sun exposure, dry skin, variable hypo- and hyperpigmented skin areas, and persistent erythematous blisters on face, since his early years. These symptoms of photosensitivity were consistent with the diagnosis of XP. Recently, he had developed an irregular, nodular and erythematous lesion on the bridge of his nose. This ulcerated, brown-black lesion measured 2 × 2 cm approximately and was suspected to be a case of malignant invasive melanoma. The haematological test showed a marked elevation in the eosinophil count (505 cells/ul), suggesting an early invasion into the underlying nasal bone. After undergoing preoperative assessment and anaesthesia eligibility test, the patient was enrolled for tumour excision and coverage via FTSG (full-thickness skin graft). The surgery was successful with adequate skin coverage on the site (Fig. 1A). The excised tumour was sent for biopsy, and the histopathology revealed an epithelioid tumour arranged in the form of nests and sheets showing melanocytic differentiation having melanin pigmentation and clear halo around the cells (Fig. 1B). After depigmentation, the cells appeared spindle to plump shaped with prominent nucleoli (Fig. 1C). The tumour was in deep dermis, with extensions into the subcutaneous fat however with tumour free resection margins. There was no evidence of lymphovascular or perineural invasion classifying the tumour stage as pT4bn1c. The patient was prescribed NSAIDs (Ibuprofen), antibiotics (amoxicillin/clavulanic acid), anti-inflammatory drugs (Danzon DS) and multivitamins post-operatively, with additional advice pertaining to wound care and minimal sun-exposure. The patient was followed up for two consecutive weeks after surgery and showed adequate recovery and no recurrence of the tumour.
